# Multiple Variations in Abdominal Aorta Branching and Their Clinical Significance: A Case Report

**DOI:** 10.7759/cureus.68256

**Published:** 2024-08-30

**Authors:** Juma Mwalimu, Kalpana Ramachandran, Kavitha Mani

**Affiliations:** 1 Anatomy, Sri Ramachandra Institute of Higher Education and Research, Chennai, IND

**Keywords:** supra- renal arteries variations, phrenic arteries variation, abdominal aorta branches variations, renal arteries variations, coeliac trunk variations

## Abstract

The abdominal aorta extends from the T12 vertebra and terminates at the L4 vertebra. It gives off anterior, lateral, and posterior branches that supply the abdominal viscera and vertebrae at this level, eventually dividing into the common iliac arteries. Dissection of the abdominal aorta and its branches from a female cadaver revealed several variations: the right inferior phrenic artery arose from the celiac trunk, the left middle suprarenal artery originated at the L1 vertebra, while the right middle suprarenal artery arose at the L2 vertebra, and the left and right renal arteries emerged from the L2 and L1 vertebrae, respectively. The gonadal arteries did not originate from the abdominal aorta. Inferior phrenic arteries may arise from the abdominal aorta, celiac trunk, or occasionally form renal arteries and are linked with extrahepatic supply in hepatocellular carcinoma. Middle suprarenal arteries typically originate from the abdominal aorta at L1, but may occasionally arise from L2 or be absent. Variations in the middle suprarenal arteries often correspond with variations in the inferior phrenic and gonadal arteries. Renal arteries may arise at the L1 vertebra, the L1/L2 intervertebral disc, or the L2 vertebra, with additional variations reported. The gonadal arteries may not originate from the abdominal aorta in some cases. These branching variations of the abdominal aorta are important for clinical, diagnostic, and therapeutic procedures and should be documented accordingly.

## Introduction

The abdominal aorta continues from the descending thoracic aorta after passing through the aortic hiatus of the diaphragm at the level of the 12th thoracic vertebra. It extends from T12 to L4 and gives rise to its terminal branches, supplying the abdominal viscera, vertebrae, and spinal cord along its course.

The abdominal aorta runs along the anterior surfaces of the T12 to L4 vertebrae, including their intervertebral discs and anterior longitudinal ligaments. It gives off ventral, lateral, posterior, and terminal branches. The ventral branches include the coeliac trunk, which originates between the lower border of T12 and the upper border of L1, the superior mesenteric artery at the L1 level, and the inferior mesenteric artery at the L3 level. The paired lateral branches consist of the inferior phrenic arteries, middle suprarenal arteries, renal arteries, and gonadal arteries. The posterior branches include four pairs of lumbar arteries and a median sacral artery. The terminal branches are the right and left common iliac arteries [1].

## Case presentation

**Figure 1:**
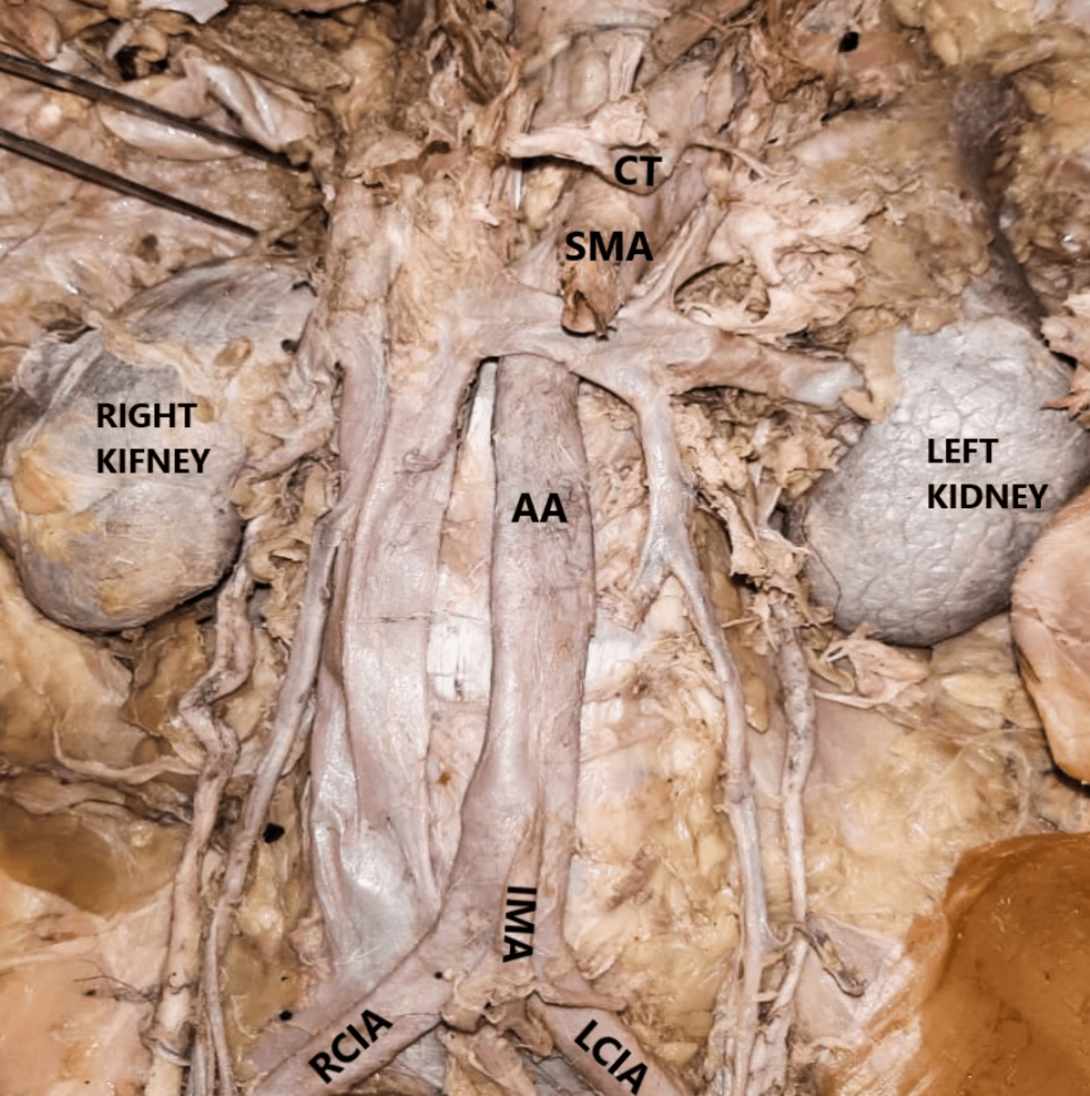
AA illustrating its ventral and terminal branches There are no gonadal arteries as part of the ventral branches of the AA. AA, abdominal aorta; CT, coeliac trunk; IMA, inferior mesenteric artery; LCIA, left common iliac artery; RCIA, right common iliac artery; SMA, superior mesenteric artery

During the routine dissection of a formalin-fixed female cadaver at Sri Ramachandra Institute of Higher Education and Research, Chennai, India, the abdominal aorta and its branches were examined (Figure 1). Various anatomical variations in the branching patterns were observed, and the details are discussed below.

**Figure 2:**
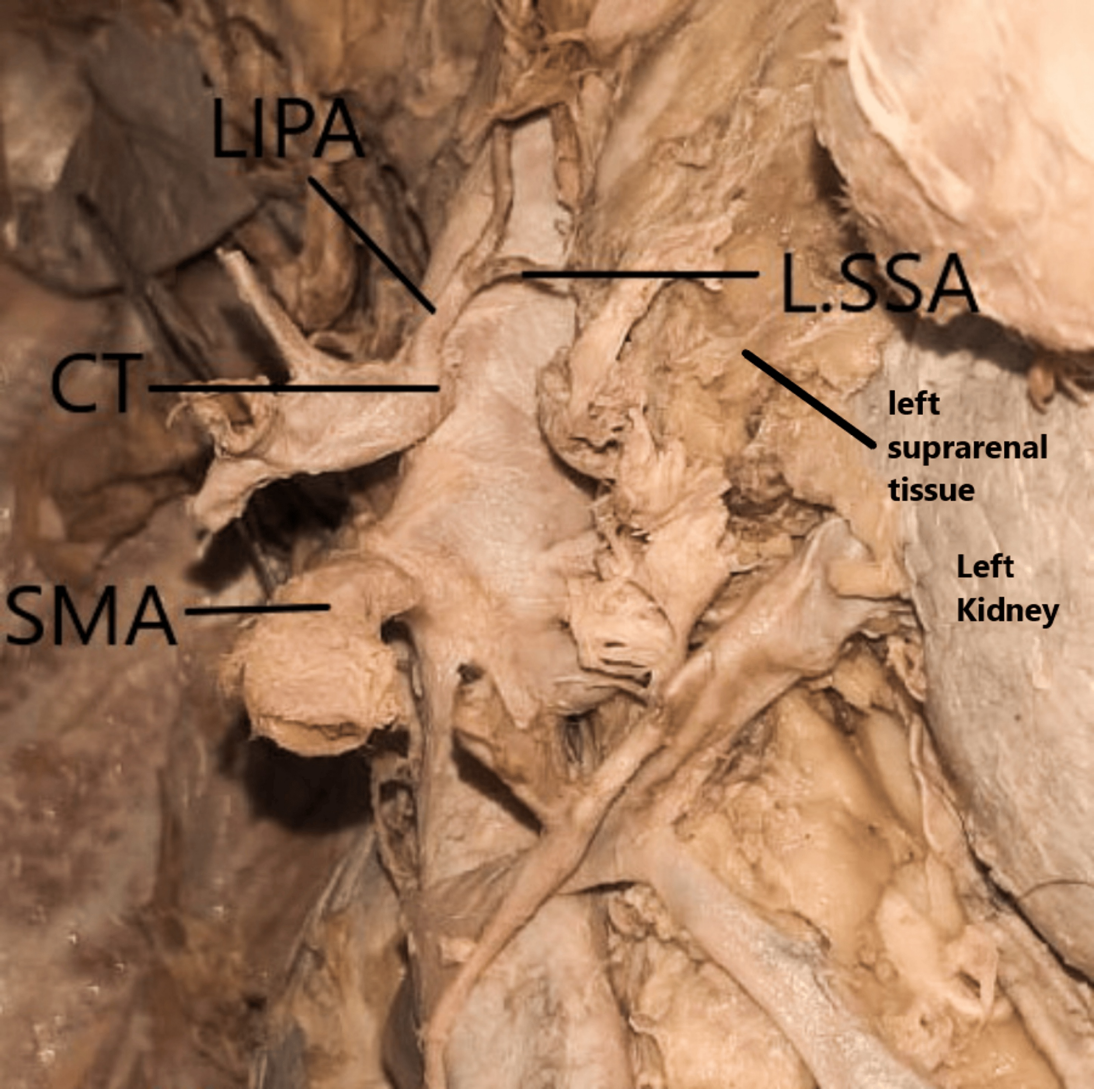
Variations in the origin of the LIPA The LIPA arises from the CT and gives off the LSSA branch. CT, coeliac trunk; LIPA, left inferior phrenic artery; LSSA, left superior suprarenal artery; SMA, superior mesenteric artery

**Figure 3:**
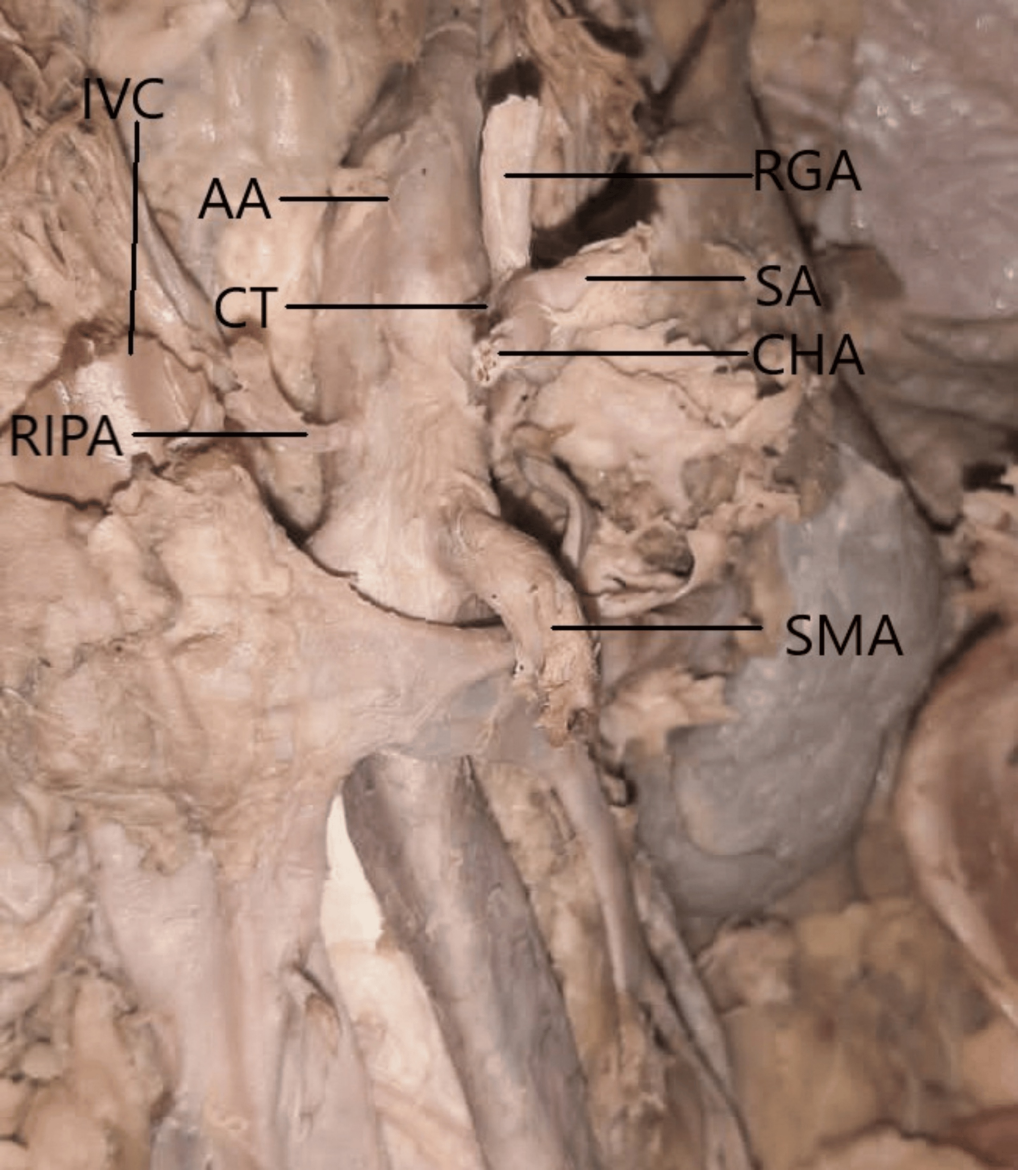
Origin of the RIPA The RIPA had a normal origin. AA, abdominal aorta; CHA, common hepatic artery; CT, coeliac trunk; RGA, right gastric artery; RIPA, right inferior phrenic artery; SA, splenic artery

The left inferior phrenic artery arose from the celiac trunk as the first branch before the celiac trunk trifurcated and then gave rise to the left superior suprarenal artery branch (Figure 2). The right inferior phrenic artery originated as a lateral branch from the abdominal aorta below the celiac trunk and above the superior mesenteric artery (Figure 3).

**Figure 4:**
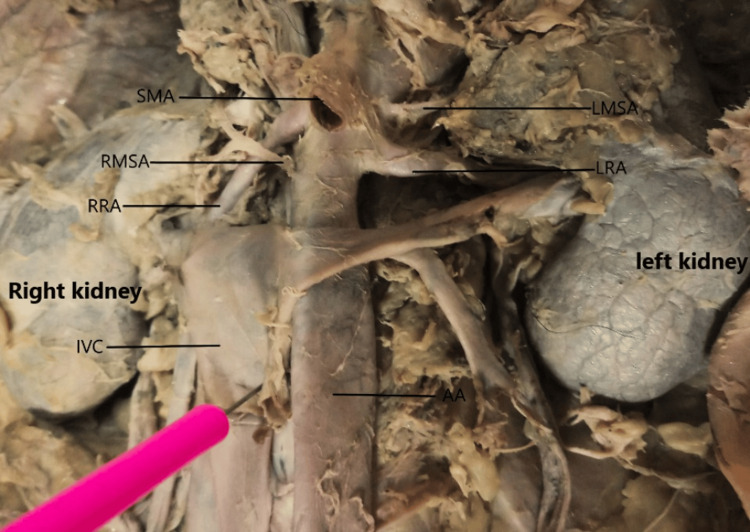
Variations in the origin of the middle suprarenal arteries and renal arteries Note that the right renal artery originates higher than the left renal artery, and the suprarenal arteries originate at the same level as the opposite renal arteries. The IVC was reflected to expose the RMSA. AA, abdominal aorta; IVC, inferior vena cava; LMSA, left middle suprarenal artery; LRA, left renal artery; RMSA, right middle suprarenal artery; RRA, renal artery; SMA, superior mesenteric artery

The right middle suprarenal artery and the left renal artery arose at the same vertebral level, specifically at the upper border of the L2 vertebra (Figure 4). The left middle suprarenal artery, superior mesenteric artery, and right renal artery originated at the same vertebral level, at the lower border of the L1 vertebra (Figure 4). Thus, the right renal artery was at the same level as the left middle suprarenal artery (L2 vertebra), and the left renal artery was at the same level as the right middle suprarenal artery (L1 vertebra). The left renal artery originated at the upper border of the L2 vertebra, which was lower than the level of the right renal artery, which originated at the lower border of the L1 vertebra (Figure 4). There were no gonadal arteries arising directly from the abdominal aorta.

## Discussion

The abdominal aorta and its branches exhibit considerable variability in their branching patterns and levels of origin. Mirjalili et al. and Pennington and Soames have reported that, in most cases, the celiac trunk arises at the T12/L1 vertebra level, the superior mesenteric artery at L1, the renal arteries at L1, the inferior mesenteric artery at L3, and the bifurcation into the common iliac arteries at L4 [1,2].

Soni and Wadhwa observed that the right inferior phrenic artery originated from the celiac trunk, while the left inferior phrenic artery originated from the abdominal aorta [3]. Loukas et al. found that both the right and left inferior phrenic arteries typically arose from the abdominal aorta just above the celiac trunk but could also originate from the celiac trunk or occasionally from the renal artery [4]. Raikos et al. noted that the left inferior phrenic artery sometimes arose from the phrenogastrosplenic trunk, which also gives rise to the left gastric artery and the splenic artery [5]. In our study, we observed that the left inferior phrenic artery originated from the celiac trunk as its first branch before the celiac trunk trifurcated, while the right inferior phrenic artery arose as a lateral branch from the abdominal aorta below the celiac trunk and above the superior mesenteric artery. Loukas et al. and Bell and Knipe highlighted the clinical importance of the inferior phrenic arteries due to their common role as an extrahepatic supply for hepatocellular carcinoma, which has implications for transcatheter embolization procedures [4,6].

Toni et al. observed that the right and left middle suprarenal arteries usually arose as lateral branches of the abdominal aorta at the level of the superior mesenteric artery [7]. In contrast, our study found that the left middle suprarenal artery originated at the level of the superior mesenteric artery, while the right middle suprarenal artery arose below the right renal artery, at the same level as the left renal artery, which is at the L2 vertebra. Priya et al. also noted the occasional absence of middle suprarenal arteries [8].

Mirjalili et al. found that the left and right renal arteries most commonly originated at the L1 vertebra in 55% and 43% of cases, respectively [1]. Mokhasi et al. reported that the renal arteries originated at L1 in 82% and 80% of cases for the left and right renal arteries, respectively [9]. Our study, however, observed that the left renal artery arose at L2 and the right renal artery at L1.

Gautam et al. reported that renal artery variations occur in about one-third of the population [10], while Aremu et al. found similar variations in 50% of individuals, with accessory renal arteries being the most common variation [11]. Mishra et al. suggested that understanding these variations is crucial for urological procedures to minimize bleeding risks and improve the treatment of renal diseases and abdominal aortic aneurysms [12].

Mokhasi et al. and Prasad et al. found that most gonadal arteries originated from the abdominal aorta, with a few originating from the renal arteries [9,13]. In our study, however, the gonadal arteries did not arise from the abdominal aorta (Table 1).

**Table 1:** Comparison of various studies on variations in the abdominal aorta branching pattern AA, abdominal aorta; CT, coeliac trunk; LIPA, left inferior phrenic artery; LRA, left renal artery; RIPA, right inferior phrenic artery; RMSA, right middle suprarenal artery

Vessel	Observation from this study	Other studies observations
LIPA	Origin from CT	From AA, CT, or renal arteries
RIPA	Origin from AA	From CT
RMSA	Origin from AA at L2 vertebra, same with LRA	From AA at L1 vertebra
LRA	Origin from AA at L2 vertebra	From AA at L1 vertebra
Gonadal arteries	Did not arise from AA	From AA

## Conclusions

From this report, we have documented multiple variations involving the abdominal aorta and their clinical significance. Notably, all the observed branching patterns from the four main branches of the abdominal aorta were present in a single cadaver. These variations underscore the importance of careful preoperative diagnostic imaging and meticulous dissection during surgeries to prevent injuries. Recognizing these variations will assist clinicians in surgical planning and improve patient outcomes. It is crucial to report such variations whenever they are encountered.
